# Increased Range of Catalytic Activities of Immobilized Compared to Colloidal Gold Nanoparticles

**DOI:** 10.3390/molecules28227558

**Published:** 2023-11-13

**Authors:** Célia Boukoufi, Ariane Boudier, Igor Clarot

**Affiliations:** 1 Université de Lorraine, CITHEFOR, F-54000 Nancy, France; celia.boukoufi@univ-lorraine.fr (C.B.); ariane.boudier@univ-lorraine.fr (A.B.); 2 Pharmacy Department, University Hospital, F-54511 Vandoeuvre-Lès-Nancy, France; 3 Institut Universitaire de France (IUF), F-75231 Paris, France

**Keywords:** nanozyme, nanostructured surface, gold nanoparticle, oxidoreductase-like activities

## Abstract

Gold nanoparticles (AuNPs) can be described as nanozymes, species that are able to mimic the catalytic activities of several enzymes, such as oxidase/peroxidase, reductase, or catalase. Most studies in the literature focus on the colloidal suspension of AuNPs, and it is obvious that their immobilization could open the doors to new applications thanks to their increased stability in this state. This work aimed to investigate the behavior of surfaces covered by immobilized AuNPs (iAuNPs). Citrate-stabilized AuNPs (AuNPs-cit) were synthesized and immobilized on glass slides using a simple dip coating method. The resulting iAuNPs were characterized (surface plasmon resonance, microscopy, quantification of immobilized AuNPs), and their multi-enzymatic-like activities (oxidase-, peroxidase-, and catalase-like activity) were evaluated. The comparison of their activities versus AuNPs-cit highlighted their added value, especially the preservation of their activity in some reaction media, and their ease of reuse. The huge potential of iAuNPs for heterogeneous catalysis was then applied to the degradation of two model molecules of hospital pollutants: metronidazole and methylene blue.

## 1. Introduction

For a long time, bulk gold has been considered without significant catalytic activity. If this is true in this state, with the era of nanotechnologies, this conviction has been questioned. It is now well-known that gold nanoparticles (AuNPs) present outstanding catalytic activities [1,2]. This property was initially studied to catalyze reactions, such as the oxidation of carbon monoxide [3] and organic molecules [4]. More recently, it was stated that NPs can act as artificial species mimicking enzymes, at the origin of the “nanozyme” concept [5]. Nanozymes are of huge interest today because of their numerous advantages compared to classical enzymes: they are cheaper, more stable, and, consequently, not limited to mild conditions of temperature, pressure, and pH. AuNPs can mimic several enzymes (multi-enzymatic activity), depending essentially on the pH of the reaction medium. At low pH (<5), the oxidase/peroxidase-like activities predominate, while at high pH (>9), it is the catalase-like activity that dominates [6]. AuNPs are also described to mimic glucose oxidase, reductase, and superoxide dismutase, and, according to their chemical surface functionalization, nuclease, esterase, or silicatein-like activities [7]. The ability of an AuNP to mimic the activity of several enzymes makes it a good model to study the impact of immobilization on the nanozyme-like activities of NPs.

Actually, most of these activities have been evaluated on colloidal suspension of AuNPs, and very little information is available about nanostructured surfaces. Gold nanostructured surfaces via AuNP immobilization (iAuNP) are active surfaces, more stable than colloidal NPs. Although colloidal AuNPs aggregate as the function of the reaction environment (solvent, pH, ionic strength), aggregation is a usual problem when working with colloidal NPs. The NP immobilization on surfaces is a common and simple solution to overcome this limitation, allowing enhanced stability over a wide range of pH or ionic strength [8]. It is already known that AuNPs keep their antioxidant [9] and catalytic activities after immobilization [10]. Indeed, our team demonstrated the ability of iAuNPs to catalyze both oxidation and reduction reactions [10].

To the best of our knowledge, the full potential of iAuNPs as nanozyme surfaces has not been yet evaluated. The studies available in the literature about the nanozyme-like activity of immobilized AuNPs mainly focus on either reductase [11,12] or catalase-like [13] activities. This work aims to investigate the whole enzyme-mimicking potential of iAuNPs and compare it with those of colloidal AuNPs. The oxidase, peroxidase, catalase, and superoxide dismutase activities will be investigated. Then, iAuNPs will be applied for the degradation of two model molecules of hospital pollutants to highlight their potential in the treatment of wastewater.

Water pollution is a global problem impacting human health, aquatic life, and ecosystem balance. Among the most polluted waters, one can cite hospital wastewater, which is contaminated with pathogens and a huge amount of pharmaceutically active compounds in comparison with domestic or municipal water. Hospital wastewater requires specific treatment, and the actual solutions are not fully satisfactory, leading to the development of new methodologies [14]. Metronidazole (MTZ) is an example of pollutants. It is an antibacterial and antiparasitic active ingredient largely excreted into the environment because of its poor biodegradation [15]. Traditional water treatment methods do not eliminate antibiotics [16]. Methylene Blue (MB) is another well-known model dye pollutant, but it is also a fluorescent dye with multi-targeted therapeutic effects used in clinical medicine [17]. It has several applications, such as an antidote (for methemoglobinemia and cyanide poisoning) and a marker and indicator in various surgical techniques and visualization of organs, and it is also used to treat dermatological diseases [18]. 

In recent years, the catalytic and nanozyme properties of nanoparticles have been widely studied as innovative and complementary tools for the degradation of pollutants [19,20]. However, the use of colloidal NPs for water treatment requires time-consuming and expensive extra steps to remove them. IAuNPs have the advantage of being easily removed from the reaction media and are reusable. Moreover, AuNPs are not devoid of toxicity [21]. Their immobilization also aims to limit their environmental propagation and consequently restrict the negative health and environmental impact of AuNPs. In this work, after the physicochemical characterization of iAuNPs and the exploration of their nanozyme activity, we applied the capability of iAuNPs to catalyze both oxidation and reduction to the degradation of these two model molecules of hospital pollutants: MTZ and MB.

## 2. Results and Discussion

### 2.1. Characterization of Surfaces with Immobilized Gold Nanoparticles

**Figure 1:**
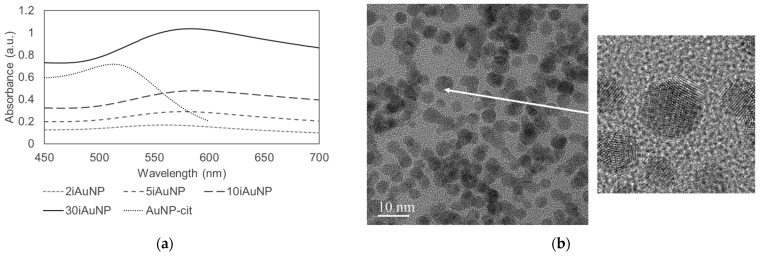
Physicochemical characterization by spectrophotometry of AuNPs-cit, 2, 5, 10, and 30iAuNPs (**a**) and a TEM picture of AuNPs-cit (**b**).

The concentration of AuNPs-cit was determined at 58 ± 8 nM, and their surface plasmon resonance (SPR, maximum wavelength) was measured at λ_max_ = 512 ± 1 nm (Figure 1a). Hydrodynamic and core diameters were 6.2 ± 0.9 nm (polydispersity index = 0.19 ± 0.08) and 2.7 ± 0.9 nm (Figure 1b), respectively, and the surface charge was measured at −25 ± 8 mV. All these physicochemical parameters were in concordance with our previously published results [10,22].

AuNPs were immobilized on the surface of glass slides with a dip coating method using a first layer of branched PEI, a polycationic polymer. Branched PEI has four pKa values (3.3, 6.7, 9.2, and 9.9 at 25 ± 0.1 °C), is positively charged at pH = 7.4 [23] and can establish electrostatic bindings with the negatively charged NP. As a function of the number of baths, the color of surfaces was clearly modified. Indeed, the SPR peak on the UV-visible spectra confirmed that AuNPs remained at the individualized state, i.e., without aggregates (Figure 1a and Table 1). However, the value of the SPR drastically increased with the number of baths (Table 1), reflecting the decrease in the distance between each particle [24] and the increase in the number of neighbors of each AuNP. Indeed, Shimizu and their team demonstrated that the SPR of Au/Al_2_O_3_ (5.1–5.5 nm) in nanocomposite films increased from 557 to 632 when the average interparticle distance decreased from 5.7 to 3.7 nm. The maximal red shift observed in this study was 74 nm, which is in concordance with the red shift between AuNPs-cit and iAuNPs, suggesting, therefore, that each nanoparticle has a neighbor closer when immobilized compared to the colloidal state. The number of neighbors of each AuNP was also proved by the increasing quantity of deposited AuNPs (up to ca. 1.6 × 10^14^ total AuNPs after 30 deposits). Concerning the oxidation state of the AuNP in the nanostructure, a complete reduction in Au^3+^ in Au^0^ has already been demonstrated for AuNPs stabilized by citrate ions at both colloidal [25] and immobilized states [26].

### 2.2. Enzyme-Mimicking Activity

#### 2.2.1. Catalase-like Activity

The reaction studied here is written in Equation (1):

2H_2_O_2_ → 2H_2_O + O_2_ (1)

**Figure 2:**
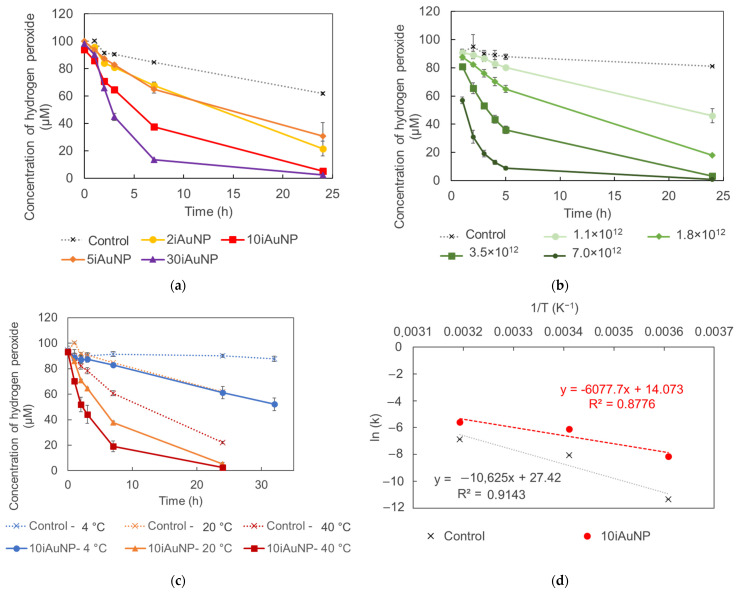
Concentrations of H_2_O_2_ at 20 °C under visible light in contact (control) with 2, 5, 10, or 30iAuNPs (**a**) or with increasing concentrations of AuNPs-cit, (**b**) (mean for *n* = 3 with corresponding standard deviation) the activity of 10iAuNPs as a function of the temperature, (**c**) and the resulting the Arrhenius equation (**d**) of the decomposition of this reaction in the presence (control) of 10iAuNPs (mean for *n* = 3 with corresponding standard deviation).

The catalase-like activity of AuNPs has already been described [6]. This activity is linked to a change in the oxidation states of metal atoms on the surface of the NP [27]. In our experiments, AuNPs-cit and iAuNPs were able to catalyze this reaction (Figure 2a,b), while PEI-coated glass slides showed no activity. 

The catalase-like activity of 10iAuNPs was evaluated at 4 ± 1 °C, 20 ± 1 °C and 40 ± 1 °C (Figure 2c,d). After 7h, this activity was calculated to be 9.3 ± 1.8%, 55.6 ± 1.5%, and 49.2 ± 4.9% for 4 ± 4 °C, 20 ± 1 °C, and 40 ± 1 °C, respectively. The temperature has already been described as an extrinsic parameter modulating the activity of the nanozyme. For example, in 2017, Ye and team showed that the peroxidase and oxidase-like activity of bimetallic metal–organic framework (MOF) (Co/2Fe) increased from 22 to 37 °C to reach a maximum and then decreased from 37 to 57 °C [28]. The impact of the temperature for the same nanozyme can be different from one enzyme-like activity to another. Luo et al. evaluated the influence of the temperature on the activity of a Ce-BPyDC (cerium-2,2′-bipyridine-5,5′-dicarboxylic acid) MOF. They revealed a maximum oxidase-like activity at 25 °C vs. 40 °C for the peroxidase-like activity [29].

The results of the catalase-like activity of 10iAuNPs according to the temperature allowed the calculation of the energy of activation (E_A_) and the pre-exponential factor (A) of the reaction (Figure 2d and Table 2). The E_A_ was reduced by more than 40% when 10iAuNPs were added compared to the control, proving that the mechanism of action in the reaction included a catalytic activity. The value of A drastically decreased. Factor A is proportional to the frequency of collisions, leading to the formation of the products of the reaction, and in this case, due to the immobilization of AuNPs, this result was expected.

However, a dose–dependent effect was also found for AuNPs-cit and iAuNPs (Figure 2a,b), which can be in contradiction with a truly catalytic activity. In general, a catalytic activity occurs with a concentration of a catalyst, which is negligible compared to substrates, and without consumption of the catalyst during the reaction. The impact of the concentration of AuNPs on their reactivity has already been described [30,31] raising questions about the real catalytic nature of AuNPs and nanozyme activities [32]. Moreover, the non-modification of the NP surface during the nanozyme reaction is also uncertain. Cafun, et al. demonstrated a charge delocalization and a modification in the valence electron of colloidal ceria NPs during catalase mimetic activity [33].

**Figure 3:**
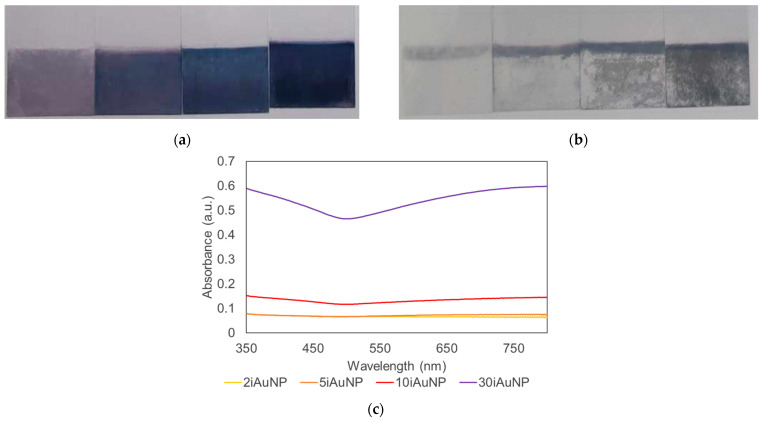
Pictures of (from left to right) 2, 5, 10, and 30iAuNPs after the immobilization (**a**) and after 24 h of storage at pH = 9.5, 20 °C, in contact with H_2_O_2_ and light for the evaluation of their catalase-like activity (**b**) and their corresponding absorbance spectra after 24 h (**c**).

Nevertheless, after 24 h in the conditions of the reaction (pH = 9.5 and in contact with H_2_O_2_), the visual aspect of iAuNPs was completely altered (Figure 3a,b). The residual absorbance was in connection with the initial amount of AuNPs immobilized on the surfacel for example, 2iAuNPs ended colorless, whereas 30iAuNPs were the most colorful iAuNPs at the end of the reaction. The absence of a real SPR may prove the modification of the NP (Figure 3c).

#### 2.2.2. Oxidase-like Activity

**Figure 4:**
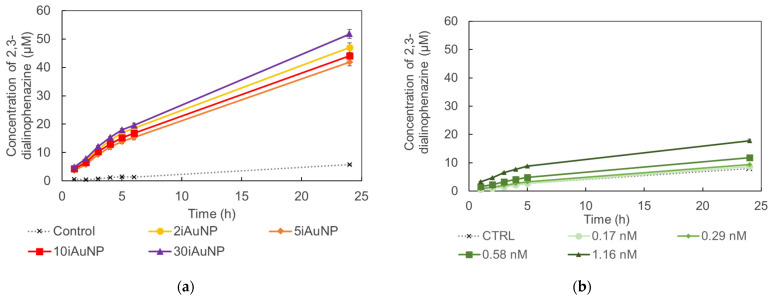
Formation of oxidized 2,3-dialinophenazine from a solution of OPDA and H_2_O_2_ in the presence (controls) of 2, 5, 10, or 30iAuNPs (**a**) or AuNPs-cit (**b**) (mean for *n* = 3 with corresponding standard deviation).

Figure 4 shows the oxidase-like activity of iAuNPs and AuNPs-cit on the oxidation of OPDA in 2,3-dialinophenazine, according to Equation (2):
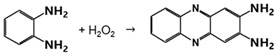
(2)

The analysis of the oxidation efficiency over time demonstrated that iAuNPs were more efficient catalysts than AuNPs-cit, even if the latter were in a lower overall number compared to iAuNPs (Figure 4, Table 3). PEI-coated glass slides showed no activity. For iAuNPs (Table 3), the oxidase-like activity was the same whatever the number of AuNP baths. On the contrary, an increased concentration of AuNPs-cit led to a visible aggregation, limiting their activity and explaining the data obtained in Table 3 for AuNPs-cit. In that sense, Zhang et al. described an extremely short activity of Au nanozymes in the oxidase activity due to the instability of AuNPs [34].

#### 2.2.3. Superoxide Dismutase-like Activity

In the studied reaction, at pH > 7, pyrogallol degrades and forms O_2_^•−^, inducing its autooxidation in purpurogallin (Equation (3)):
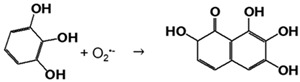
(3)

**Figure 5:**
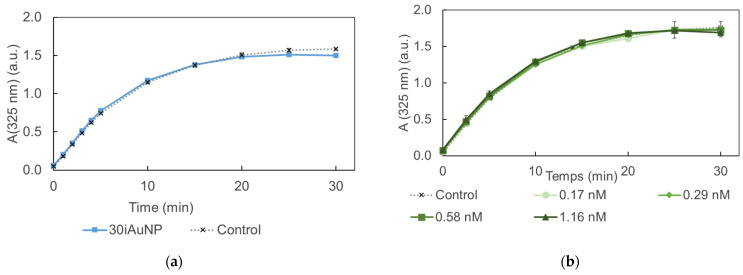
Evolution of the formation of purpurogalline from pyrogallol at 25 °C in contact (control) with 30iAuNPs (**a**) or AuNPs-cit (**b**) (mean for *n* = 3 with corresponding standard deviation).

The results presented in Figure 5 show an absence of activity for both conditions (i.e., iAuNPs and AuNPs-cit). The SOD-like activity of 10iAuNPs was −1.8 ± 1.1% and 30iAuNPs was 5.3 ± 1.5%, even though the SOD-like activity of AuNPs has already been described in the literature. However, in their study, Yin and his team worked with AuNPs coated with polyvinylpyrrolidone or tannic acid [35]. It is well known that surface functionalization has a huge impact on the nanozyme activity of AuNPs [36]. Moreover, tannic acid is known to give a positive answer to a SOD-like assay. Another study described the SOD-like activity of AuNPs in combination with copper NPs [37]. To the best of our knowledge, no SOD-like activity has been described for AuNPs-cit. 

#### 2.2.4. Peroxidase-like Activity

From TMB, the reaction leads to ox-TMB, as illustrated in Equation (4):
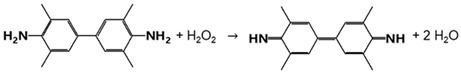
(4)

**Figure 6:**
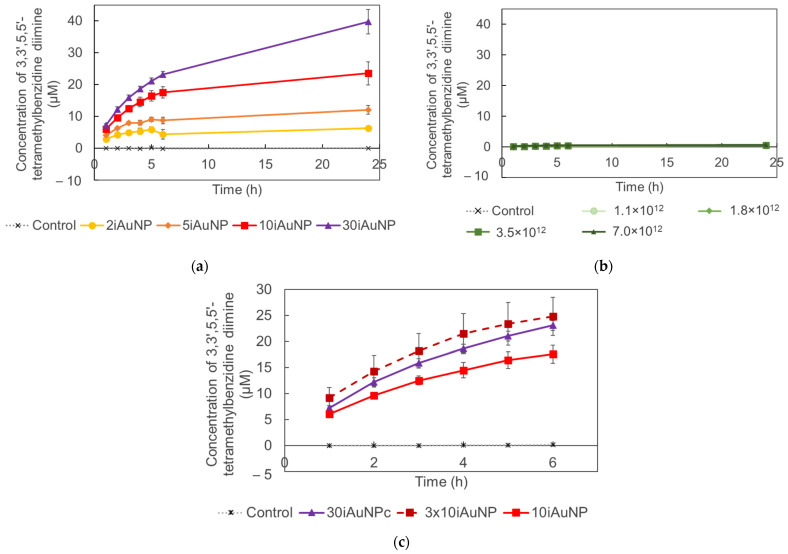
Formation of oxidized TMB from a solution of TMB in the presence (control) of iAuNPs (**a**) or AuNPs-cit and (**b**) comparison of the activity of 10iAuNPs, 30iAuNPs, and three 10iAuNPs dipped simultaneously (**c**) (mean for *n* = 3 with corresponding standard deviation).

The results in Figure 6 proved that contrary to AuNPs-cit, iAuNPs showed a dose–dependent peroxidase-like activity (Figure 6a), while PEI-coated glass slides showed no activity. The absence of activity observed in the case of AuNPs-cit (Figure 6b) was explained by their precipitation in the reaction medium. TMB is very poorly soluble in water and must be dissolved in dimethyl sulfoxide before use. At the colloidal state, AuNPs were not stable in these conditions. Their immobilization increased their stability and allowed them to take advantage of their peroxidase-like activity.

Some may think that the multiple rinses during the immobilization process may lead to the elimination of the citrate ions at the surface of AuNPs, leaving the gold core immobilized on the surface without any citrate ions. Indeed, Lin and his team synthesized unmodified/bare AuNPs that were stable for two months by reducing HAuCl_4_ with NaBH_4_ as the sole reducing agent without a stabilizing agent. They showed that bare AuNPs presented increased peroxidase-like activity than citrate-capped AuNPs [38]. This hypothesis could explain the difference in activity observed between AuNPs-cit and iAuNPs. However, Boujday’s team immobilized AuNPs on silicon surfaces. The monitoring of the surface using surface-enhanced Raman scattering indicated the persistence of a citrate ion on the surface after immobilization [39]. This study disproved the previous hypothesis, and the presence of peroxidase-like activity only in the case of iAuNPs should be due to their higher stability regarding the reaction media. 

We also compared the peroxidase-like activity of 30iAuNPs to three 10iAuNP slides incubated together (Figure 6c). For the first 6 h, no significant difference was observed (k30iAuNP = 3.10 ± 0.12 µM·h^−1^ and k3x10iAuNP = 3.11 ± 0.75 µM·h^−1^). However, at 24 h the activity of 30iAuNPs increased and was superior compared to the three surfaces of 10iAuNPs. Both were more active than a unique 10iAuNP (k10iAuNP = 2.29 ± 0.40 µM·h^−1^). As mentioned in Table 1, 10.1 ± 0.2 × 10^13^ and 15.7 ± 0.2 × 10^13^ are the total numbers of AuNPs immobilized at the surface of 10 and 30iAuNPs, respectively. Three 10iAuNP slides incubated together represented a total of 30.3 × 10^13^ AuNPs added in the reaction media or twice that of 30iAuNPs. The higher activity of the three slides of 10iAuNPs compared to the one slide of 10iAuNPs indicated that the number of AuNPs in contact with the TMB solution was a critical factor for peroxidase-like activity. However, the overall increased activity of 30iAuNPs highlighted the importance of the organization of AuNPs on the glass slide. This organization can lead to more or less AuNP surface available for the reaction, which is known to be proportional to its reactivity [40].

#### 2.2.5. Successive Catalysis Reactions

**Figure 7:**
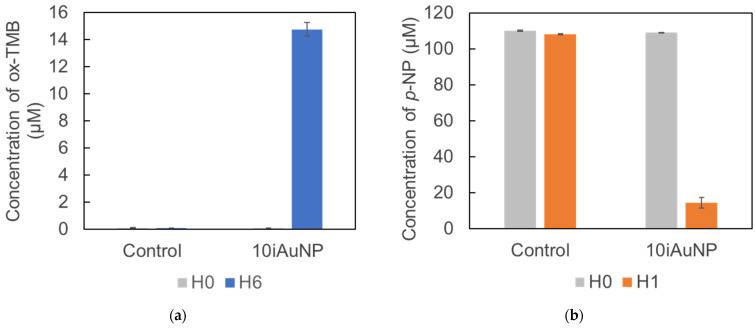
Concentration of formed ox-TMB after 6 h in the presence (control) of 10iAuNPs (peroxidase-like activity) (**a**) and a residual concentration of p-NP after 1 h in the presence (control) of the same 10iAuNPs (reductase-like activity) (**b**) observed during the successive catalysis reactions.

In this part of the study, 10iAuNPs were used for two successive catalytic reactions. First, the peroxidase-like reaction during 6 h was performed, and then the reductase-like one was performed for the same duration. In a previous work, a deep study of the reductase activity of iAuNPs was demonstrated [10]. The 10iAuNP showed the ability to catalyze successively the peroxidation of TMB and had a reduction in *p*-NP (Figure 7). 

On the one hand, when the 10iAuNPs were first used for the peroxidation of TMB, the total concentration of ox-TMB formed after 6 h was 14.8 ± 0.5 µM (Figure 7a). The reductase activity was then evaluated at 86.7 ± 2.7%, corresponding to a reduction of 94.7 ± 3.0 µM of *p*-NP in 1 h (Figure 7b).

In an attempt, we tried to realize first the reductase activity but due to the harsh experimental conditions (NaBH_4_ 50 mM), the material was degraded, as seen by the modification of the colored surface, implying that the second reaction could not be tested.

In this experiment, 10iAuNPs have been successfully reused to catalyze, peroxidadize, and then reduce reactions. To the best of our knowledge, the ability of immobilized AuNPs to catalyze successively oxidation and reduction is described for the first time. 

Colloidal AuNPs have already been described in the literature as reusable catalysts. In one study, AuNPs were removed by the centrifugation of AuNPs embedded in polyaniline composite nanospheres, and polyaniline acted as a dispersing agent and stabilized AuNPs to avoid their agglomeration [41]. In another study, AuNPs dispersed in fibrous silica microspheres with γ-Fe_2_O_3_ magnetic cores were removed using a magnet [42]. Indeed, once added to a reaction medium, colloidal NPs cannot be easily recovered. Traditional methods for separating colloidal NPs are often time-consuming and require high energy or expensive equipment. Most of these methods involve aggregation, with separation often achieved by long or high-speed ultracentrifugations [43]. It can also be induced by the addition of a stimulus leading to the precipitation of NPs, such as modification of the ionic charge [43] or UV exposure [44]. 

Because the precipitation leads to modification or loss of NP activity, it limits the possibility of reuse and recycling. This highlighted the added value of iAuNPs over colloidal AuNPs. The nanostructured surface can be easily removed from a reaction media, rinsed, and soaked in another one. These results corroborated the reusability study of iAuNPs already conducted by our team in which iAuNPs were used to catalyze reduction reactions more than 20 times over 130 days [10].

### 2.3. Degradation of Pollutants

#### 2.3.1. Degradation of Metronidazole

We investigated the capability of iAuNPs to degrade a pollutant. MTZ is an antibacterial and antiparasitic active ingredient found in a significant way in the environment. One way to degrade MTZ is a reduction, for example, in the presence of NaBH_4_, leading to the formation of several well-described degradation products [45,46]. We already described the reductase-like ability of iAuNPs. The same property was used to catalyze the degradation of MTZ.

**Figure 8:**
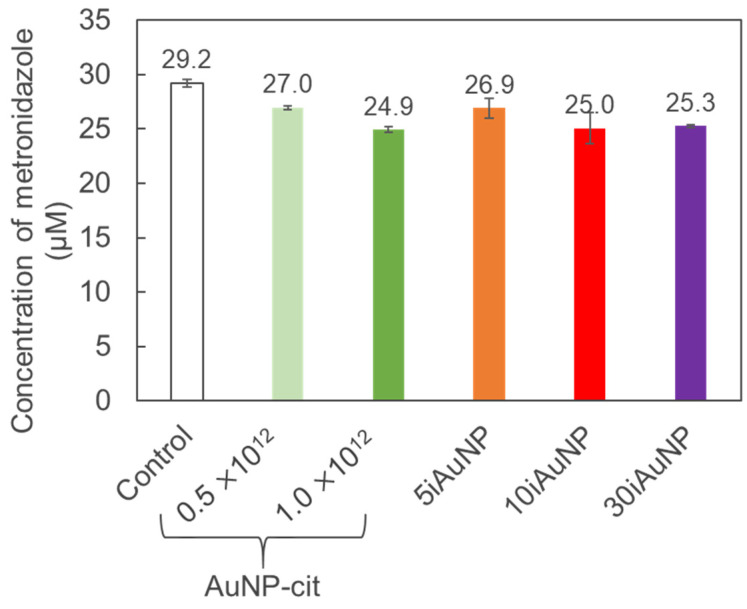
Concentration of MTZ after 10 min of degradation in contact (control) with AuNPs-cit or 5, 10, or 30iAuNPs and NaBH4 at 20 °C and 150 rpm stirring (mean for *n* = 3 with corresponding standard deviation).

Both AuNPs-cit and iAuNPs were able to catalyze this degradation under reductive conditions (Figure 8 and Table 4). The 10iAuNP (100.7 ± 1.7 × 10^12^ total number of immobilized AuNP) had the same activity as the 1.10 × 10^12^ AuNP-cit. This difference has already been described and was justified by the reduction in the Brownian motion and the availability of their surface electron after immobilization [13,15]. 

The activity of iAuNPs increased from 5 to 10iAuNPs but was the same for 10 and 30iAuNPs (Table 4). An important increase in all the nanozyme-like activity has been described when the number of deposits of AuNPs goes from 2 or 5 to 10. This improvement was less marked from 10 to 30 deposits, even for the number of AuNPs immobilized at the surface of glass slides. 

#### 2.3.2. Degradation of Methylene Blue

IAuNPs are able to catalyze both reduction and oxidation reactions. Consequently, we investigated the ability of iAuNPs to degrade hospital pollutants under oxidative conditions, using MB as an example [47]. Several colloidal NPs are described in the literature as able to catalyze the degradation/discoloration of MB in the presence of H_2_O_2_, such as TiO_2_-NPs [48] and Nb_2_O_5_-NPs [49]. In both cases, the discoloration of MB was induced by the activation of H_2_O_2_ in reactive oxygen species, leading to the oxidative degradation of MB.

**Figure 9:**
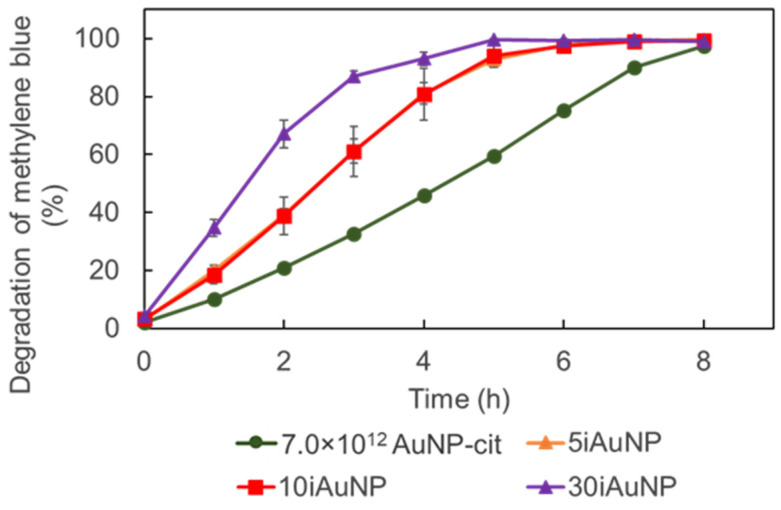
Degradation activity of 7.0 × 10^12^ AuNPs-cit and 5, 10, or 30iAuNPs over 8 h at 20 °C and 150 rpm stirring (mean for *n* = 3 with corresponding standard deviation); please note that the curves for 5 and 10iAuNPs overlap.

Both AuNPs-cit and iAuNPs were able to catalyze the degradation of MB under oxidative conditions (Figure 9). Unlike the degradation of MTZ, for this reaction, iAuNPs were more effective with a faster kinetic than the highest quantity of tested AuNPs-cit (7.0 × 10^12^ AuNP). The 30iAuNP achieved a complete discoloration (>99%) in 6 h (degradation activity was 99.4 ± 0.3%), and 5 and 10iAuNPs required 7 h (degradation activities were 99.2 ± 0.3 and 99.1 ± 0.5%, respectively), while after 8 h, the degradation activity of AuNPs-cit was 97.5 ± 0.5%. One possible explanation for the difference in efficacy of iAuNPs and AuNPs-cit in the degradation of MTZ and MB is the nature of reactions. MTZ was degraded using reduction whereas MB was degraded by oxidation. As demonstrated earlier, iAuNPs appeared to have an increased reactivity compared to AuNPs-cit for oxidation reactions. On the other hand, we have already demonstrated that immobilization reduces the kinetic of AuNPs-cit in the case of reductase-like activity [10].

## 3. Materials and Methods

### 3.1. Reagents and Standards

All reagents and solvents were of analytical grade, and they were used without further purification. Potassium phosphate monobasic was purchased from Acros Organics, (Geel, Belgium) hydrogen peroxide was from VWR (AnalaR Normapur, Bruchsal, Germany), hydrochloric acid (1 M) was from Carl Roth (Karlsruhe, Germany), and sodium bicarbonate was from Cooper (Bergenfield, NJ, USA). All the other reagents were purchased from Sigma-Aldrich (St. Louis, MO, USA). Ultrapure deionized water (>18.2 MΩ cm) was used for the preparation of all solutions. The glass slides were 24 × 36 mm borosilicate coverslips purchased from Menzel-Gläzer (Bremen, Germany).

### 3.2. Preparation of Gold-Nanostructured Glass Slides (iAuNPs)

#### 3.2.1. Synthesis and Characterization of Citrate-Stabilized Gold Nanoparticles

Citrate-stabilized AuNPs (AuNPs-cit) were synthesized in the laboratory according to [18]. Briefly, in 90 mL of ultrapure water, 1 mL of a HAuCl_4_ aqueous solution (25 mM), 2 mL of a sodium citrate aqueous solution (55 mM), and 1 mL of NaBH_4_ dissolved in a sodium citrate solution (19.5 mM) were successively incorporated. The reaction took place under N_2_ and stirring (250 rpm). The citrate-stabilized AuNPs were stored at +4 °C for a maximum of 20 days [22] and used without further purification.

The characterization of AuNPs included the record of their UV–visible spectra for the measurement of their absorbance (A_AuNP_) (UV-2600 Shimadzu, Kyoto, Japan), the determination of their hydrodynamic diameter in volume mode (D_h_), and their zeta potential (ζ) at 25 ± 1 °C with Zetasizer Nano ZS (Malvern Instruments, Malvern, UK). Their concentration (C_AuNP_ (mol·L^−1^) was calculated according to Lambert–Beer’s law (ε_AuNP_ = 1.2 × 10^7^ M^−1^·cm^−1^ (taken at the surface plasmon resonance (SPR) band (maximum wavelength) [50]. Transmission electron microscopy (TEM) investigations were carried out using a JEM-ARM 200F Cold FEG TEM/STEM operating at 200 kV on a copper grid.

#### 3.2.2. Immobilization of Citrate-Stabilized Gold Nanoparticles

AuNPs were immobilized with a method previously described [9]. Briefly, glass slides were cleaned and activated with two 15 min immersions at 100 °C, first in an aqueous solution of sodium dodecyl sulfate (0.1 M) and then in an HCl solution (0.1 M). Finally, pre-activated glass slides were rinsed with distilled water. 

**Figure 10:**
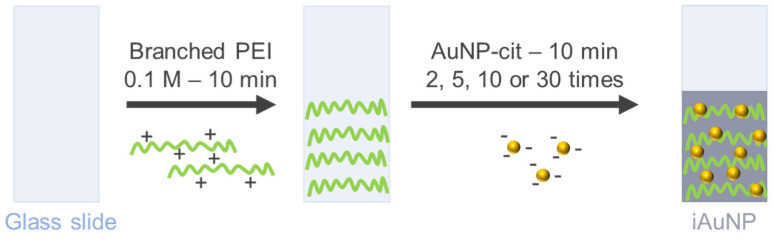
Schematic diagram of AuNP immobilization.

A Tris−HCl buffer was obtained by dissolving 18.2 g of Trizma^®^ base in 1 L of ultrapure water (0.15 M), and pH was adjusted to pH = 7.4 with HCl 12 M. A 0,1 M polymer solution was prepared by dissolving branched polyethyleneimine (PEI) in a Tris−HCl buffer. The activated glass slides were soaked for 10 min in the PEI solution and were immersed in a bath of AuNPs-cit for 10 min 2, 5, 10, or 30 times (corresponding to 2, 5, 10, or 30-iAuNPs, respectively) (Figure 10). Each bath was followed by a rinse step with a Tris−HCl buffer (3 min) and a soak in water. The iAuNPs were stored at +20 °C, and we demonstrated their activity over 130 days [10].

#### 3.2.3. Characterization of Immobilized Gold Nanoparticles

The nanostructured glass slides were characterized using a UV-vis spectrophotometer (UV-2600 Shimadzu). For the Ion Beam Scanning Electron Microscope (FIB-SEM) (Helios Nanolab 600i and FEI brand), the surface of the nanostructured glass slide was directly observed without preliminary metallization. The ion-emitting gallium source was set to 20 kV. Energy dispersive X-ray spectroscopy (EDS) was performed on a part of the nanostructured glass slide.

Quantification of immobilized AuNPs was carried out using an Inductively Coupled Plasma Optical Emission Spectroscopy (ICP-OES) method. Briefly, nanostructured glass slides were first crushed and mineralized in contact with 1 mL of H_2_O, 5 mL of nitric acid 65%, and 1 mL of hydrochloric acid 37%. The samples were then digested using a Microwave Digestion System (Multiwave GO, Anton Paar, Tokyo, Japan) for 50 min at 185 °C. The solution was recovered, and the volume was adjusted to 10.0 mL with a 1% nitric acid aqueous solution. In parallel, a calibration range of HAuCl_4_ from 0.0125 to 0.5000 mg·L^−1^ in 1% nitric acid was realized. The solutions were injected using a peristaltic pump (set at 45 rpm) in the ICP system (ICP-OES, iCAP Pro, ThermoScientific, Waltham, MA, USA) with argon as auxiliary gas. The nebulizing pressure was 2.1 bar, and the flow rates of the nebulizer and cooler were 0.5 L·min^−1^ and 12.5 L·min^−1^, respectively. Data acquisition was performed using Qtera software (version 2) at a working wavelength of 242.795 nm.

### 3.3. Enzyme-Mimicking Activities

Each activity was investigated for AuNPs-cit (30, 50, 100, and 200 µL of 60 nM AuNPs-cit, corresponding to a final number of 1.1, 1.8, 3.5, and 7.0 × 10^12^ AuNPs) and iAuNPs (2, 5, 10, and 30iAuNPs). The amount of AuNPs-cit used for each reaction corresponded to the amount required to observe a significative effect. Moreover, at this concentration, the absorbance of AuNPs-cit did not interfere with the spectrophotometric measurement.

#### 3.3.1. Catalase-like Activity

A solution of hydrogen peroxide in a 15 mM carbonate/bicarbonate buffer pH = 9.5 (100 µM) was stored in contact (control) with AuNPs-cit or iAuNPs, 24 h at 20 ± 1 °C under visible light. The concentration of H_2_O_2_ was followed with a spectrophotometric measurement at 240 nm (ε = 43.6 M^−1^·cm^−1^ [51]) after a ten-fold dilution in the buffer. To evaluate the influence of the temperature on the activity of iAuNPs, the catalase-like activity of 10iAuNPs was also investigated at 4 ± 4 °C (in a cold room) and 40 ± 1 °C (using a thermostatic water bath). For each temperature, the constant of kinetic (k) for the reaction with and without 10iAuNPs was calculated using a first-order rate law using Equation (5). Given the stability of iAuNPs at several temperatures and the reaction kinetics, k at 4 °C was calculated at 32 h and 7 h for 20 and 40 °C.

C = C_0_·e^−kt^ (5)

where C is the concentration of hydrogen peroxide at a specific time t, C_0_ is the initial concentration, and k is the constant of kinetic.

Then, the activation energy (E_A_) and the pre-exponential factor (A) were determined using the Arrhenius equation (Equation (6)):

Ln(k) = ln A·(E_A_/(R × T)) (6)

where R is the universal gas constant (8.314 J·K^−1^·mol^−1^) and T is the the temperature in Kelvin.

In parallel, the catalase-like activity was calculated using Equation (7):

Catalase-like activity (%) = (C_0_ − C_1_)/C_0_ × 100 (7)

where C_0_ is the concentration of hydrogen peroxide in the control and C_1_ is the concentration of the sample.

#### 3.3.2. Oxidase-like Activity

Forty microliters of a 0.1 M o-phenylenediamine (OPDA) solution in HCl 0.1 M were added with 200 µL of hydrogen peroxide aqueous solution (20 mM) and a 15 mM phosphate buffer, pH = 4.5, for a final volume of 10.0 mL. The final solution was stored in contact (control) with AuNPs-cit or iAuNPs, for 24 h at 40 °C (water bath), under stirring, protected from light. The oxidase-like activity was evaluated by monitoring the formation of the yellow oxidation product of OPDA, 2,3-diaminophenazine (DAP), with a spectrophotometric measurement at 449 nm (the molar absorption coefficient was calculated at ε = 20,500 M^−1^·cm^−1^). The oxidase-like activity was calculated using Equation (8):

Oxidase-like activity (%) = (C_0_ − C_1_)/C_0_ × 100 (8)

where C_0_ is the concentration of the control at 5 h and C_1_ is the concentration of the sample.

#### 3.3.3. Superoxide Dismutase-like Activity

One hundred microliters of a 2 mM pyrogallol in solution in HCl 100 µM were added in 9.9 mL of a Tris-EDTA-HCl buffer (50 mM, 10 mM), pH = 8.2. The final solution was stored in contact (control) with AuNPs-cit or iAuNPs, for 30 min at 25 ± 2 °C (using a thermostatic bath), under stirring, protected from light. The superoxide dismutase-like (SOD) activity was evaluated by monitoring the formation of the yellow oxidation product of pyrogallol, the purpurogalline, with a spectrophotometric measurement at 325 nm. The SOD-like activities of AuNPs-cit and iAuNPs were calculated using Equation (9):

SOD-like activity (%) = (A_0_ − A_1_)/A_0_ × 100 (9)

where A_0_ is the absorbance of the control at 325 nm at 30 min and A_1_ is the absorbance of the sample.

#### 3.3.4. Peroxidase-like Activity

3,3′,5,5′-tetramethylbenzidine (TMB) was dissolved in dimethylsulfoxide at a concentration of 4.2 mM. One milliliter of this solution was added to 4 µL of a hydrogen peroxide aqueous solution 30% (*v*/*v*) and a 15 mM phosphate buffer, pH = 4.5, for a final volume of 10.0 mL. The solution was stored in contact (control) with AuNPs-cit or iAuNPs, for 24 h at 20 ± 1 °C under visible light. The peroxidase-like activity was evaluated by monitoring the formation of the blue oxidized 3,3′,5,5′-tetramethylbenzidine (ox-TMB) with a spectrophotometric measurement at 652 nm (ε = 39,000 M^−1^·cm^−1^ [52]).

The same protocol was carried out to investigate the impact of the surface architecture of iAuNPs. Consequently, the peroxidase-like activity of 30iAuNPs and those of three 10iAuNP slides incubated together were compared. The kinetic constants (k), were calculated, on the 6 first hours, using a first-order rate law using Equation (5): where C is the concentration of ox-TMB at t time and C_0_ is the initial one.

#### 3.3.5. Study of Successive Catalysis 

10iAuNPs were used for their peroxidase-like and immediately after for their reductase-like activity. The peroxidase-like activity was evaluated with the experimental conditions described in the previous part. The total amount of ox-TMB formed was measured at 0 and 6 h. The glass slide was then rinsed with ultrapure water and used to measure the reductase-like activity. This activity was evaluated using the reduction of *p*-nitrophenol (*p*-NP) to *p*-aminophenol (*p*-AP) at 20 ± 2 °C. Five milliliters of 0.1 mM aqueous *p*-NP and 5 mL of 0.1 M aqueous NaBH_4_ were used 10iAuNPs were directly dipped in the 10 mL reagent solutions and stirred at ca. 150 rpm. Catalytic performance was followed using a UV−Vis spectrophotometer (UV-2600 Shimadzu). The consumption of the *p*-NP was measured at 400 nm after 1 h and calculated using Beer–Lambert’s law with ε = 9.19 × 10^3^ L·mol^−1^·cm^−1^. The reductase-like activity was calculated using Equation (10):

Reductase-like activity (%) = (C_0_ − C_1_)/C_0_ × 100 (10)

where C_0_ is the concentration of *p*-NP in the control and C_1_ is the concentration of *p*-NP in the sample after 1 h.

### 3.4. Degradation of Pollutants

#### 3.4.1. Degradation of Metronidazole

To evaluate the ability of iAuNPs to catalyze the degradation of MTZ, 250 µL of MTZ (1.0 mM) dissolved in HCl 1.0 M were mixed with 5.0 mL of NaBH_4_ (5.0 mM) and 4.75 mL of a 0.148 M phosphate buffer saline solution (PBS), pH = 7.4. The PBS solution was prepared by dissolving 6.68 mM of Na_2_HPO_4_, 1.47 mM of KH_2_PO_4_, 138.00 mM of NaCl, and 2.68 mM of KCl = 2.68 mM in ultrapure water with a final pH adjusted to 7.4 with HCl 1.0 M.

The previous MTZ solution was incubated in contact with or without (control) AuNPs-cit (0.5, 1.0, or 1.7 × 10^12^ AuNPs) or with 5, 10, and 30iAuNPs at 20 ± 1 °C under visible light and stirring (150 rpm). After the reaction was completed (10 min), the absorbance was read at 320 nm (molar absorption coefficient ε_MTZ_ = 8730 M^−1^·cm^−1^ [53]). The reduction efficiency was calculated using Equation (11):

Degradation efficiency (%) = (C_0_ − C_1_)/C_0_ × 100 (11)

where C_0_ is the concentration of the control at 10 min and C_1_ is the concentration of the sample.

#### 3.4.2. Degradation of Methylene Blue

To evaluate the ability of iAuNPs to catalyze the degradation of MB, nine milliliters of a solution of MB in tap water was stored in contact with 1 mL of H_2_O_2_ 30% (*v*/*v*), for a final concentration of MB of 30 µM. The solution was stored in contact with or without (control) AuNPs-cit (1.7 × 10^12^ AuNPs) or with 5, 10, and 30iAuNPs. The solutions were incubated at 20 ± 1 °C under visible light and stirring (150 rpm). The absorbance was read at 663 nm at each hour for a total of 8 h to follow the discoloration of MB. The degradation efficiency was calculated using Equation (12):

Degradation efficiency (%) = (A_0_ − A_1_)/A_0_ × 100 (12)

where A_0_ is the absorbance of the control and A_1_ is the absorbance of the sample.

## 4. Conclusions

In this work, AuNPs-cit were successfully immobilized on the surface of glass slides, and we demonstrated their multi-enzymatic-like activity. IAuNPs presented both oxidase, peroxidase, and catalase-like activity. The nanozyme activities were compared to AuNPs-cit. This comparison illustrated the added value of iAuNPs, which offer an increased stability compared to AuNPs-cit in several reaction media. This work also highlighted the complex mechanism of action of AuNPs-cit and iAuNPs as nanozymes and some factors influencing their activity. The addition of iAuNPs reduced the EA required for the degradation of hydrogen peroxide, but their activity cannot be fully described according to a catalytic model, mainly due to the concentration-dependent effect. A better understanding of this mechanism of action seems essential to increase the predictability of AuNPs-cit- and iAuNP-induced reactions. The reusability in certain conditions of iAuNPs and their potential to catalyze the degradation of pollutants (such as MTZ and MB) could be applied to offer new opportunities in the treatment of hospital wastewater, such as the formation of flow-through catalytic reactors for various heterogeneous catalysis.

## Figures and Tables

**Table 1:** UV-visible spectroscopic characterization of iAuNPs and the total number of deposited AuNPs as a function of the number of baths (mean of *n* = 3 with corresponding standard deviation).

Sample	Number of AuNP-Cit Baths	SPR Value (nm)	Amax (at SPR)	Total Number of Deposited AuNPs
2iAuNP	2	559 ± 4	0.168 ± 0.004	3.8 ± 0.1 × 10^13^
5iAuNP	5	587 ± 3	0.298 ± 0.008	7.5 ± 0.3 × 10^13^
10iAuNP	10	585 ± 4	0.504 ± 0.016	10.1 ± 0.2 × 10^13^
30iAuNP	30	585 ± 1	1.102 ± 0.044	15.7 ± 0.2 × 10^13^

**Table 2:** Physicochemical parameters of the decomposition of hydrogen peroxide in water and dioxygen in the presence of 10iAuNPs (control) determined using Arrhenius’ law, (mean for *n* = 3 with corresponding standard deviation).

Condition	Ea (kJ·mol^−1^)	A (s^−1^)
Control	88.4 ± 9.8	1.1 ± 1.7 × 10^13^
10iAuNP	50.6 ± 5.8	6.9 ± 0.1 × 10^6^

**Table 3:** Oxidase-like activity of 2, 5, 10, or 30iAuNPs and AuNPs-cit (mean for *n* = 3 with corresponding standard deviation).

State	Number of AuNPs	Reduction Efficiency (%)
AuNP-cit	1.1 × 10^12^	−1.8 ± 1.8
	1.8 × 10^12^	19.6 ± 4.7
	3.5 × 10^12^	75.6 ± 5.5
	7.0 × 10^12^	220.8 ± 6.3
2iAuNP	38.1 ± 1.3 × 10^12^	1070.8 ± 54.9
5iAuNP	75.4 ± 3.2 × 10^12^	856.2 ± 47.9
10iAuNP	100.7 ± 1.7 × 10^12^	949.4 ± 52.5
30iAuNP	156.8 ± 1.9 × 10^12^	1159.6 ± 9.7

**Table 4:** MTZ reduction efficiency of AuNPs-cit and iAuNPs after 10 min at 20 °C and 150 rpm stirring in the presence of NaBH_4_ (mean for *n* = 3 with corresponding standard deviation).

State	Number of AuNPs	Reduction Efficiency (%)
AuNP-cit	0.5 × 10^12^	7.7 ± 0.6
	1.0 × 10^12^	14.6 ± 0.8
5iAuNP	75.4 ± 3.2 × 10^12^	8.8 ± 3.0
10iAuNP	100.7 ± 1.7 × 10^12^	15.1 ± 4.8
30iAuNP	156.8 ± 1.9 × 10^12^	14.4 ± 0.4

## Data Availability

Data are contained within the article.
